# Efficient SABRE-SHEATH Hyperpolarization of Potent Branched-Chain-Amino-Acid Metabolic Probe [1-^13^C]ketoisocaproate

**DOI:** 10.3390/metabo13020200

**Published:** 2023-01-29

**Authors:** Isaiah Adelabu, Md Raduanul H. Chowdhury, Shiraz Nantogma, Clementinah Oladun, Firoz Ahmed, Lukas Stilgenbauer, Marianna Sadagurski, Thomas Theis, Boyd M. Goodson, Eduard Y. Chekmenev

**Affiliations:** 1 Department of Chemistry, Integrative Biosciences (Ibio), Karmanos Cancer Institute (KCI), Wayne State University, Detroit, MI 48202, USA; 2 Department of Chemistry, Department of Physics, Joint UNC-CH & NC State Department of Biomedical Engineering, North Carolina State University, Raleigh, NC 27695, USA; 3 School of Chemical & Biomolecular Sciences and Materials Technology Center, Southern Illinois University, Carbondale, IL 62901, USA; 4 Russian Academy of Sciences, Leninskiy Prospekt 14, 119991 Moscow, Russia

**Keywords:** NMR, hyperpolarization, carbon-13, ketoisocaproate, signal amplification by reversible exchange, SABRE-SHEATH, branched chain amino acid, BCAT

## Abstract

Efficient ^13^C hyperpolarization of ketoisocaproate is demonstrated in natural isotopic abundance and [1-^13^C]enriched forms via SABRE-SHEATH (Signal Amplification By Reversible Exchange in SHield Enables Alignment Transfer to Heteronuclei). Parahydrogen, as the source of nuclear spin order, and ketoisocaproate undergo simultaneous chemical exchange with an Ir-IMes-based hexacoordinate complex in CD_3_OD. SABRE-SHEATH enables spontaneous polarization transfer from parahydrogen-derived hydrides to the ^13^C nucleus of transiently bound ketoisocaproate. ^13^C polarization values of up to 18% are achieved at the 1-^13^C site in 1 min in the liquid state at 30 mM substrate concentration. The efficient polarization build-up becomes possible due to favorable relaxation dynamics. Specifically, the exponential build-up time constant (14.3 ± 0.6 s) is substantially lower than the corresponding polarization decay time constant (22.8 ± 1.2 s) at the optimum polarization transfer field (0.4 microtesla) and temperature (10 °C). The experiments with natural abundance ketoisocaproate revealed polarization level on the ^13^C-2 site of less than 1%—i.e., one order of magnitude lower than that of the 1-^13^C site—which is only partially due to more-efficient relaxation dynamics in sub-microtesla fields. We rationalize the overall much lower ^13^C-2 polarization efficiency in part by less favorable catalyst-binding dynamics of the C-2 site. Pilot SABRE experiments at pH 4.0 (acidified sample) versus pH 6.1 (unaltered sodium [1-^13^C]ketoisocaproate) reveal substantial modulation of SABRE-SHEATH processes by pH, warranting future systematic pH titration studies of ketoisocaproate, as well as other structurally similar ketocarboxylate motifs including pyruvate and alpha-ketoglutarate, with the overarching goal of maximizing ^13^C polarization levels in these potent molecular probes. Finally, we also report on the pilot post-mortem use of HP [1-^13^C]ketoisocaproate in a euthanized mouse, demonstrating that SABRE-hyperpolarized ^13^C contrast agents hold promise for future metabolic studies.

## 1. Introduction

NMR has enabled a wide range of applications, one of the most important of which is MRI. Conventional biomedical MRI relies on the nuclear spin polarization (*P*) of protons (typically of water and fat) for visualization of organ morphology, structure, and function. In clinical MRI scanners, *P* is low (*P*_H_ = 10^−5^ or 0.001%), which significantly limits the overall sensitivity. As a consequence, only compounds at a high physiological concentration such as water protons (ca. 100 M) [1] can be detected easily. Imaging of lower-concentration species is challenging, and only possible at the expense of spatial and temporal resolution [2].

NMR hyperpolarization enables transient *P* increases, up to order unity [1,3,4,5] (i.e., 100%). Hyperpolarized (HP) exogenous contrast agents can be administered via inhalation or injection [1,6,7,8,9,10,11]. The hyperpolarized signals are increased by 4–5 orders of magnitude [12], and enable the use of a relatively small bolus of HP contrast agent [13,14] for enhanced MRI. For example, a human dose of HP [1-^13^C]pyruvate only requires ~1 g [15], and a mouse dose only ~1–3 mg [16]. The use of ^13^C-hyperpolarized contrast agents, instead of ^1^H agents, has garnered increasing attention because the ^13^C nucleus (despite being less MR-sensitive than ^1^H) offers several advantages [17,18]. First, the ^13^C nucleus of carboxylate moieties can retain the HP state for minutes in vivo [17], in sharp contrast to ^1^H, where polarization decays in seconds. Second, ^13^C has a negligible background signal [17]. Third, carbon chains form the backbone of biomolecules, and thus, the ^13^C nucleus can be engineered into a versatile and sensitive biomolecular probe [17]. Fourth, carboxylate moieties in particular offer a wide range of ^13^C chemical shift dispersion [17], which is useful for differentiating downstream metabolic products of injected HP ^13^C molecular probes [17]. These combined advantages allow for real-time metabolic imaging of exogenous ^13^C HP contrast agents [19,20].

[1-^13^C]pyruvate is currently the most developed contrast agent due to its central role in cellular energy pathways [17,18,21]. It is now under evaluation in over 30 clinical trials according to clinicaltrials.gov. Other HP ^13^C metabolic probes are being developed to selectively image corresponding metabolic pathways, which may be upregulated in a wide range of diseases—most notably in cancer [18].

[1-^13^C]ketoisocaproate is structurally similar to [1-^13^C]pyruvate. Both of them are α-ketocarboxylates with ketoisocaproate having a longer side chain compared to [1-^13^C]pyruvate: -CH(CH_3_)_2_ instead of –CH_3_. This structural difference has a profound impact on the biochemical utilization of HP [1-^13^C]ketoisocaproate in vivo. The pioneering work by Karlsson and co-workers demonstrated the biochemical conversion of injected HP [1-^13^C]ketoisocaproate to HP [1-^13^C]leucine [22]. This transformation is catalyzed by the enzyme, branched-chain amino acid transferase (BCAT) [22]. BCAT is a marker for cancer metastasis and a target of the proto-oncogene c-myc [22]. Karlsson and co-workers demonstrated that metabolic conversion is greatly elevated in murine lymphoma (EL4) metastatic cells compared to surrounding tissues and other tumor cell types (more than 7-fold difference) [22]. In addition to the utility for molecular imaging of cancer demonstrated by Karlsson and co-workers, Butt and co-workers have also demonstrated that HP [1-^13^C]ketoisocaproate may be a promising HP contrast agent for evaluation of cerebral BCAT activity, which is dysregulated in neurodegenerative disease [23,24]. These pioneering studies [22,23] have utilized the dissolution dynamic nuclear polarization (d-DNP) hyperpolarization technique to produce HP [1-^13^C]ketoisocaproate for in vivo studies.

**Scheme 1:**
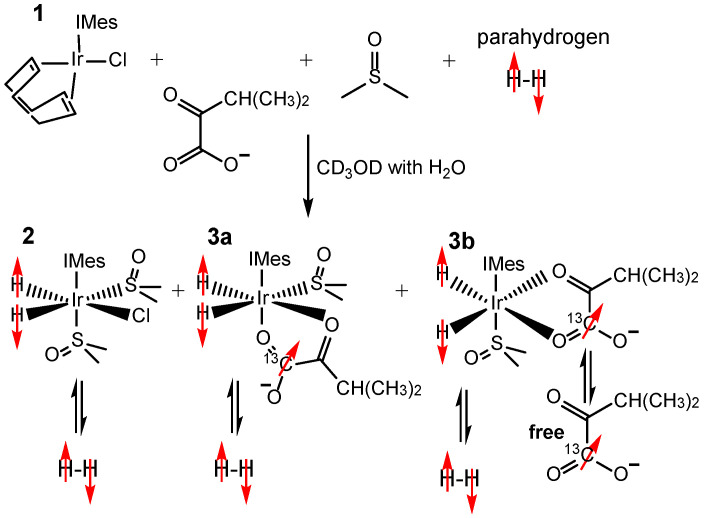
Chemistry of activation of pre-catalyst (**1**) and formation of complexes **2, 3a,** and **3b**. Adapted from previous assignments by Duckett and co-workers [28].

The d-DNP technique requires the use of cryogenic equipment and high-field magnets to hyperpolarize substrate molecules, resulting in high cost and low throughput of d-DNP instrumentation [12,25,26,27]. Recently, Tickner and coworkers demonstrated that [1-^13^C]ketoisocaproate can be hyperpolarized via SABRE-SHEATH (Signal Amplification By Reversible Exchange in SHield Enables Alignment Transfer to Heteronuclei) [28]. SABRE-SHEATH [29,30] relies on the simultaneous exchange of parahydrogen (p-H_2_) and a to-be-hyperpolarized substrate with a coordinating metal complex [31] in the presence of a sub-microtesla magnetic field [32]. The use of an IrIMes polarization transfer catalyst [33] with co-ligating DMSO (Scheme 1) leads to the formation of the SABRE-active complex **3b** that enables coordination of a α-ketocarboxylate in the equatorial plane [34,35]. During the transient coordination of ketoisocaproate, the spin order of p-H_2_-derived hydrides is spontaneously transferred to the ^13^C nucleus [28]. The equatorial positions of the IrIMes catalyst [33] shown in Scheme 1 are exchangeable, leading to the release of HP ketoisocaproate. Multiple cycles of substrate exchange led to a build-up of “**free**” (i.e., not catalyst-bound) HP ketoisocaproate. Unlike in **3b**, in **3a** the ketoisocaproate substrate ligates via an axial position, rendering ketoisocaproate virtually non-exchangeable on the time scale of the SABRE-SHEATH NMR experiment (at least several seconds to a minute or more), Scheme 1. Tickner and co-workers demonstrated *P*_13C_ of up to 0.8% for [1-^13^C]ketoisocaproate [28].

Here, we report on several key advances of SABRE-SHEATH hyperpolarization of [1-^13^C]ketoisocaproate to significantly improve hyperpolarization levels. First, through optimization of temperature, and the use of higher p-H_2_ pressure in our p-H_2_ bubbling apparatus, we demonstrate the feasibility of *P*_13C_ of over 18% [1-^13^C]ketoisocaproate, representing a >20-fold *P*_13C_ increase over the pioneering report [28]. Second, we have also investigated the feasibility of SABRE-SHEATH hyperpolarization of natural abundance (1.1% ^13^C) ketoisocaproate, which allows us to study ^13^C polarization of C-1 and C-2 independently. These studies reveal dramatically reduced levels of ^13^C polarization of the C-2 site compared to C-1, which is in part due to less favorable relaxation and in part due to less efficient *J*-coupling and less favorable exchange rates. Third, this work reports on the influence of pH, which reveals a remarkable modulation of the SABRE-SHEATH polarization chemistry. Fourth, the substantially higher SABRE-SHEATH *P*_13C_ level reported here enabled dynamic magnetic resonance spectroscopy (MRS) in a mouse post-mortem: HP [1-^13^C]ketoisocaproate was injected in a recently euthanized animal to demonstrate that HP [1-^13^C]ketoisocaproate can be detected inside an animal using a 0.35 T clinical MRI scanner. Combined with recent developments describing bio-compatible formulations of ^13^C contrast agents polarized by SABRE-SHEATH [36], these results pave the way to future in vivo studies.

## 2. Materials and Methods

### 2.1. Hyperpolarizer Setup

**Scheme 2:**
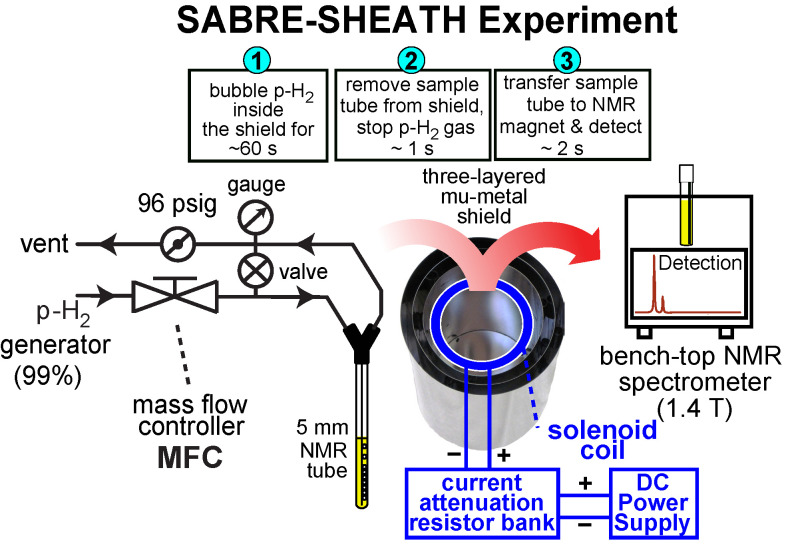
Experimental schematic of the SABRE-SHEATH hyperpolarizer equipped with a benchtop 1.4 T NMR spectrometer (SpinSolve Carbon, Magritek, Wellington, New Zealand). Parts of this scheme are adapted with permission from Ref. [40]. Copyright 2018 American Chemical Society.

The hyperpolarizer apparatus contains a three-layered mu-metal shield equipped with a degaussing coil and circuit [37]. This setup (to be described in full detail elsewhere) is also equipped with a solenoid magnet inside the inner mu-metal in series with an attenuating resistor bank and 5 VDC power supply to provide precise control of the magnetic field inside the shield, Scheme 2 [38]. The field was monitored by a fluxgate magnetometer, and p-H_2_ gas flow was metered by a mass flow controller (MFC). Temperature control of the sample was maintained using a water bath, Scheme 2 **[38]**. The p-H_2_ over-pressure (over atmospheric value) is achieved using a 96 psig valve to provide ~8 bar of total p-H_2_ pressure inside the sample tube, Scheme 2. Access to p-H_2_ is provided via a p-H_2_ storage and distribution setup described in detail previously [39]. Full details including the bill of materials (BOM) for the hyperpolarizer setup will be presented in the near future in a separate publication.

### 2.2. Sample Preparation for SABRE Hyperpolarization

All experiments employed the standard SABRE pre-catalyst for SABRE [IrCl(COD)(IMes)] (IMes = 1,3-bis(2,4,6-trimethylphenyl)imidazol-2-ylidene; COD = cyclooctadiene; species **1**, Scheme 1) [33,41,42], which was synthesized as described previously [43]. For SABRE experiments, a solution containing 5 mM SABRE pre-catalyst **1**, 30 mM sodium ketoisocaproate, 20 mM dimethylsulfoxide (DMSO, AC348445000, Fisher Scientific, Waltham, MA, USA) in CD_3_OD (DLM-24-25, Cambridge Isotope Laboratories Inc., Tewksbury, MA, USA) was prepared. Both natural abundance sodium ketoisocaproate (K0629, Sigma-Aldrich) or sodium [1-^13^C]ketoisocaproate (99% ^13^C, 487716, Sigma-Aldrich, St. Louis, MO, USA) were used as received. A 0.6-mL aliquot of the prepared solution was transferred into a medium-wall 5 mm NMR tube (Wilmad-LabGlass, P/N 504-PP-9), which was equipped with ¼” outer-diameter (OD) and 3/16” inner diameter (ID) Teflon tube jacket extension (50–80 mm long, McMaster Carr, Elmhurst, IL, USA, P/N 5239K12). This Teflon tubing allows connecting the NMR tube to the push-to-connect wye connector (McMaster Carr, Elmhurst, IL, USA, P/N 5779K262) of the p-H_2_ bubbling manifold, Scheme 2 [37].

Then, the sample was immediately purged with ultra-high-purity argon gas (>99.9999%) to remove any trapped air by bubbling pure argon through the solution for at least 1 min and capped with a precision rubber cap (Sigma-Aldrich, St. Louis, MO, USA, P/N Z554014-100EA). The prepared sample was stored at room temperature for 0.2–2 h prior to SABRE hyperpolarization studies. Next, the tube was connected to the hyperpolarizer setup via a ¼”-push-to-connect adapter, Scheme 2. Bubbling of p-H_2_ through the sample was established via 1/16” OD Teflon tubing (1/32” inner diameter (ID), McMaster Carr, Elmhurst, IL, USA, P/N 5239K23), Scheme 2. The bubbling tube was equipped with an optional Teflon tube insert (0.9 mm OD, 0.5 mm ID); this thinner catheter minimized susceptibility-induced magnetic field gradients, and therefore, improves the field homogeneity (and subsequently full width at half maximum (FWHM) and sensitivity) of HP molecules’ detection for SABRE studies. Note the narrower catheter also delivers smaller p-H_2_ bubbles potentially increasing surface-to-volume ratio for gas exchange. Closing or opening the valve shown in Scheme 2 results in p-H_2_ bubbling through the sample or cessation of the bubbling, respectively. Each sample was activated for at least ten minutes prior to SABRE studies with ultra-high-purity (>99.999%) p-H_2_ (>98% para-state [44,45]) at a flow rate of 70 standard cubic centimeters (sccm), Scheme 2. The sample activation leads to the removal of the COD moiety via hydrogenation [46] and formation of the hexa-coordinate species as shown in Scheme 1.

### 2.3. NMR Signal Detection

Following cessation of p-H_2_ bubbling, the sample containing the HP material was transferred to a 1.4 T benchtop NMR spectrometer (transit time: ~2 s), where the ^1^H or ^13^C NMR spectra were recorded, Scheme 2. The corresponding NMR signals were integrated using Prospa software provided by the spectrometer vendor. The integrated values were either employed directly to analyze the polarization trends (e.g., build-up, decay, etc.), or these values were converted to ^13^C signal enhancement (*ε*_13*C*_) and *P*_13*C*_ values in accord with Equations (1) and (2) below.

### 2.4. ^13^C NMR Signal Enhancement (ε_13C_) and Polarization (P_13C_) Calculations

The level of signal enhancement was calculated using the concentration-based signal-referencing equation proposed by Shchepin et al. by integrating the data obtained from the 1.4 T benchtop Magritek Spinsolve NMR spectrometer [47], using the following equation.

(1) $$\varepsilon_{13C}=\frac{S_{HP}}{S_{REF}}\times \frac{C_{REF}}{C_{HP}}\times \frac{A_{REF}}{A_{HP}}$$

where *S_REF_* and *S_HP_* denote the NMR signals for a neat thermally polarized reference sample ([1-^13^C]acetic acid) and HP ketoisocaproate samples, respectively. *C_REF_* and *C_HP_* represent the concentrations of the thermally polarized [1-^13^C]acetic acid signal–reference sample (17.5 M) and the concentration of the HP ketoisocaproate sample (30 mM). The *A_REF_* and *A_HP_* ratio (which corrects for differences in the effective cross-sectional areas of the HP and reference samples) was taken to be approximately 1.53 as described in previous studies [48,49] (note that the slight difference of 1.53 (this report) compared to 1.70 (previous studies) is to do narrower p-H_2_ bubbling catheter employed in this study). In some cases, we have calculated the *ε*_13*C*_ for “**free**” and the sum of **3a** + **3b** species using 25 mM and 5 mM concentrations of the “**free**” species and the pre-catalyst, respectively, and using the integrated signal intensities of the corresponding ^13^C resonances.

The percentage *P*_13*C*_ was calculated using the equation below by multiplying the equilibrium ^13^C spin polarization (1.2 × 10^−4^%) at 1.4 T and 298 K by the corresponding *ε*_13*C*_.

(2) $$P_{13C}=\varepsilon_{13C}\times 1.2\times 10^{-4}\%$$

### 2.5. ^13^C Polarization Build-Up and T_1_ Relaxation Measurements

**Figure 1:**
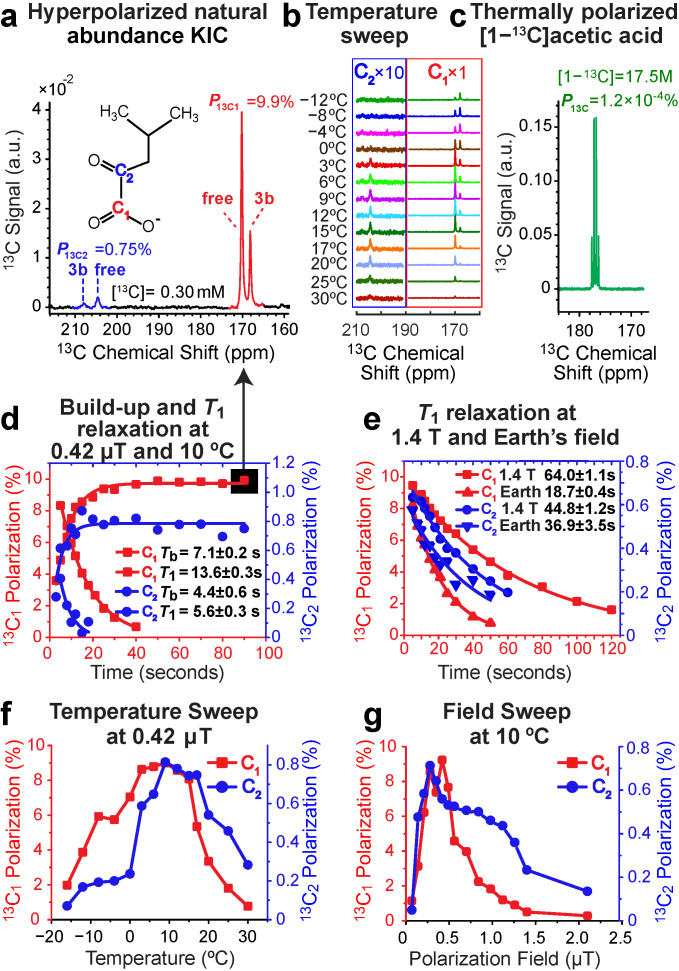
^13^C SABRE-SHEATH studies of natural abundance ketoisocaproate (KIC). (**a**) ^13^C spectrum of HP KIC. (**b**) Stacked variable-temperature ^13^C spectra of KIC showing the interplay between HP complex **3b** and the “**free**” resonances for C1 and C2 sites as a function of temperature. (**c**) Corresponding ^13^C spectrum of the thermally polarized neat [1-^13^C]acetic acid reference sample. (**d**) *P*_13*C*_ build-up and *T*_1_ relaxation at B_T_ = 0.42 μT and 10 °C. (**e**) *T*_1_ at 1.4 T and the Earth’s magnetic field; (**f**) *P*_13*C*_ temperature sweep at B_T_ = 0.42 μT; (**g**) *P*_13*C*_ polarization transfer field (B_T_) sweep at 10 °C. C1 and C2 carbon positions are color-coded by red and blue, respectively.

In each ^13^C polarization build-up experiment, the p-H_2_ bubbling time at the optimal polarization transfer field (e.g., ~0.42 µT) is varied systematically until a steady *P*_13*C*_ level is achieved. A separate experiment is employed to acquire each data point to comprise an entire build-up curve for such an experiment, e.g., Figure 1d. For ^13^C *T*_1_ relaxation measurements, p-H_2_ was bubbled for 60 s to achieve the steady-state *P*_13C_ at 0.42 µT. Next, the p-H_2_ flow was stopped, and the sample was either kept at the same field or transferred to a different magnetic field of interest (e.g., the Earth’s magnetic field of approximately 50 µT or 1.4 T field of the NMR spectrometer). Then, the sample was allowed to depolarize for a variable period of time in a systematic fashion. As with the build-up curves, a separate experiment is employed to acquire each data point comprising a given *T*_1_ decay curve, e.g., Figure 1d,e. For each experimental series, a mono-exponential build-up or decay function was fit to the experimental *P*_13*C*_ build-up or decay data using Origin Pro software (OriginLab, Northampton, MA, USA).

### 2.6. pH Modulation Study

All experiments (unless noted otherwise) were performed using the given sample composition without any pH adjustment. In some cases (as noted in the text), sample pH dependence was modified by adding 50 µL of 1 M HCl (Fisher Scientific, Waltham, MA, USA, P/N A144-212) solution in CD_3_OD to 0.6-mL sample aliquot in CD_3_OD. This addition reduced the pH value of the prepared sample from the initial value of 6.1 (unaltered sample) to 4.0 (acidified sample). The pH adjustment was performed before sample activation with p-H_2_ bubbling, and the pH was tested by a portable pH meter (P/N SX811-MS, APERA Instruments, Columbus, OH, USA).

### 2.7. Post-Mortem Studies Using HP [1-^13^C]ketoisocaproate Injection in a Euthanized Mouse

The rationale for the pilot injection of HP [1-^13^C]ketoisocaproate into a freshly euthanized mouse post-mortem was two-fold: our first goal was to demonstrate quantitative delivery of hyperpolarization payload into an animal placed inside an MRI scanner. Our second goal was to investigate the ^13^C *T*_1_ relaxation of an injected solution under (nearly) physiologically relevant conditions. The overarching goal is to validate all necessary steps prior to future utilization of HP contrast media prepared via SABRE-SHEATH in an alcohol solvent followed by reconstitution into aqueous media using a recently demonstrated approach [36]. The experimental setup consists of a replica SABRE-SHEATH hyperpolarizer (Scheme 2), and a custom-made transmit-receive circuit for ^13^C NMR spectroscopy. The circuit consists of a solenoid made from 24 American Wire Gauge (AWG) (0.8 Ω resistance, 72.8 µH inductance, 3.50 cm length, 27 mm ID, and 31 mm OD). This solenoid was tuned to the ^13^C Larmor frequency in a parallel circuit with a 1-120 pF non-magnetic trimmer capacitor (C_T_). The circuit was matched to 50 Ω impedance using a non-magnetic trimmer capacitor C_M_. The final circuit tuning and matching were performed inside a 0.35 T homogeneous MRI magnet (Pica, Time Medical, Hong Kong) using a Kea2 NMR spectrometer (Magritek, Wellington, New Zealand) equipped with a 200 W Tomco amplifier.

Prior to experiments with the post-mortem animal model, the NMR coil was calibrated, and a reference signal from thermally polarized neat [1-^13^C]acetic acid (0.0732 moles) was recorded using a 120 μs excitation RF pulse, corresponding to a tipping angle (α) of 86°. For the post-mortem experiments, a freshly euthanized 12-week-old C57BL/6 male mouse [50] (~euthanized 1h before the contrast agent injection) received two injections of HP [1-^13^C]ketoisocaproate. For each injection, a fresh batch of HP [1-^13^C]ketoisocaproate was produced using the SABRE-SHEATH polarizer, the HP liquid (~0.6 mL) was transferred into a plastic vial and loaded into a plastic syringe (1 mL) and injected intraperitoneally (i.p.) into a euthanized mouse via a 20-gauge metal needle. Approximately 0.3 mL of HP liquid was injected containing 9 × 10^−6^ moles of HP [1-^13^C]ketoisocaproate. The total time between the HP agent production (i.e., cessation of p-H_2_ flow) and the ^13^C MRS detection was ~18 s. Dynamic ^13^C MRS was performed with 2 s temporal resolution (32 total NMR spectra recorded) using α = 16.5° (23 μs). Other acquisition parameters included spectral width (SW) = 5 kHz, number of acquisition points = 2048, and acquisition time (AQ) = 409.6 ms. Once the first dynamic run was completed, another batch of HP [1-^13^C]ketoisocaproate was produced, and the injection protocol was repeated. The time delay between the start of the first and the second runs was approximately 5 min. Because the reference sample positioning (in the center of the solenoid) was similar to that of the injection location with respect to the detection coil (and because of the lack of circulation and metabolism in the euthanized animal), the following molar quantity-based approach was employed (the above-described concentration-based approach was unsuitable due to uncertainly to HP concentration after injection) to compute the ^13^C signal enhancement of the injected contrast agent of the first acquisition during dynamic MRS run:

(3) $$\varepsilon_{13C}=\frac{S_{HP}}{S_{REF}}\times \frac{N_{REF}}{N_{HP}}\times \frac{\sin{\left(\alpha \right)}_{REF}}{\sin{\left(\alpha \right)}_{HP}}$$

where *N_REF_* and *N_HP_* represent the molar quantity of the thermally polarized [1-^13^C]acetic acid signal–reference sample (0.0732 moles) and the molar quantity of HP [1-^13^C]ketoisocaproate sample (9 × 10^−6^ moles); and sin(α)*_REF_*/sin(α)*_HP_* accounts for the difference in the excitation pulse angle in signal reference and HP detection (3.4 in our case). The determined ^13^C signal enhancements were multiplied by the thermal *P*_13*C*_ (3.08 × 10^−5^%) at 0.35 T (resonance frequency of 3.81860 MHz) and 298 K:

(4) $$P_{13C}=\varepsilon_{13C}\times 3.08\times 10^{-5}\%$$

Following each injection of HP [1-^13^C]ketoisocaproate, the recorded ^13^C signal decayed due to *T*_1_ relaxation and the application of RF excitation pulses. No metabolism was anticipated, which would otherwise manifest in the appearance of additional HP resonances. To account for ^13^C depolarization due to RF excitation, the signal intensities were corrected as follows: *S_HP_*(i) = *S_HP_*(i)/[cos(α)^(i−1)^], where i is the acquisition number ranging from 1 to 32. After such compensation for *P*_13*C*_ loss due to RF pulses, the data were fitted to a model of mono-exponential decay to determine the ^13^C *T*_1_.

## 3. Results and Discussion

### 3.1. ^13^C SABRE-SHEATH of Natural Abundance Ketoisocaproate

Recently, we have demonstrated that ^13^C-hyperpolarized molecules can be comprehensively and efficiently studied at the natural abundance level of the ^13^C isotope (1.1%) using a bench-top NMR spectrometer [51], including SABRE-SHEATH-hyperpolarized compounds [39]. Such studies become possible because the detection sensitivity of lower field instruments can be nearly as good as that of the higher field spectrometers in the context of NMR hyperpolarization because the nuclear spin polarization of the HP state is not endowed by the field of the NMR spectrometer employed for the detection [52]. Studying SABRE-SHEATH hyperpolarization at natural ^13^C abundance effectively allows studying the *P*_13*C*_ polarization dynamics of each carbon independently because the probability of having two ^13^C nuclei next to each other is negligible.

For natural abundance ketoisocaproate (KIC), *P*_13*C*1_ of 9.9% and *P*_13*C*2_ of 0.80% were observed for C1 and C2 sites, respectively, Figure 1a,f. In line with the previous SABRE studies with structurally similar pyruvate and α-ketoglutarate [39,53], the exchange rate between the “**free**” and **3b** species depends on the temperature, which is evident from the ratio of the relative intensities of the HP ^13^C “**free**” and **3b** peaks of the C1 and C2 sites, Figure 1b. At elevated temperatures, the “**free**” HP resonance dominates the spectrum because fast exchange (on the time scale of the SABRE experiment) leads to effective averaging of the HP magnetization over “**free**” and **3b** species, which are present in solution in approximately a 5:1 ratio. On the other hand, at temperatures of −4 °C and below, the spectra are dominated by the catalyst-bound **3b** resonance, clearly indicating that exchange is slow on the time scale of the SABRE experiment, and the bulk of the HP magnetization is retained on the **3b** catalyst-bound species. Moreover, performing a temperature sweep experiment (shown in Figure 1f) reveals a polarization maximum at 6–8 °C for both C1 and C2 resonances. The magnetic field sweep of C1 and C2 resonances follows a slightly different trend, with *P*_13*C*_ maxima around 0.4 µT and 0.3 µT, respectively (Figure 1g). This optimal field is similar to the previously reported value for the structurally similar pyruvate [38,54] and α-ketoglutarate [39]. This observation is rationalized by the overall similar complex structures of keto-carboxylates with comparable values of spin–spin couplings relevant to the polarization transfer from p-H_2_-derived hydrides to the target ^13^C nucleus.

The *P*_13*C*_ build-up time of 7.1 ± 0.2 s corresponds to a build-up rate of ~0.14 s^−1^, which is substantially faster than the corresponding spin relaxation rate of ~0.07 s^−1^ (*T*_1_ = 13.6 ± 0.3 s) for the C1 site at 0.4 µT, Figure 1d. Because the spin relaxation rate is much slower than the build-up rate, it is kinetically possible to reach relatively high polarization values, e.g., *P*_13*C*_ of 10%. Furthermore, the long ^13^C1 *T*_1_ values of 64.0 ± 1.1 s and 18.7 ± 0.4 s at 1.4 T and the Earth’s magnetic field (Figure 1e) demonstrate good potential for the use of the C1 site as a polarization storage site for future applications. The corresponding ^13^C relaxation dynamics for the C2 site at 0.4 µT are less favorable, Figure 1d, with the *T*_b_ and *T*_1_ values, reduced to just 4.4 ± 0.6 s and 5.6 ± 0.3 s, respectively. These less favorable relaxation dynamics likely contribute to overall reduced *P*_13*C*_ values for the C2 site, yet differences in hydride target *J*-coupling and differences in the exchange processes may also contribute.

The ratio of maximum polarization values for the C1 and C2 resonances was 12:1, whereas this ratio was 12:3 for pyruvate, and 12:0 for α-ketoglutarate [39]. This observation of C2 polarization efficiency ranking (pyruvate > KIC > α-ketoglutarate) correlates well with the decrease in the corresponding side chain (-CH_3_ < -CH(CH_3_)_2_ < -CH_2_-CH_2_-COO^−^). We hypothesize that having a longer side chain can be partially responsible for less favorable dynamics during the SABRE-SHEATH hyperpolarization process. It is also anticipated that the C2 site has stronger spin–spin interactions with the chain protons, which can potentially act as relaxation sinks at low (i.e., microtesla) magnetic fields [55], thereby resulting in effectively shorter ^13^C *T*_1_ values and lower *P*_13*C*_ values as compared to the C1 site.

### 3.2. ^13^C SABRE-SHEATH of [1-^13^C]ketoisocaproate

**Figure 2:**
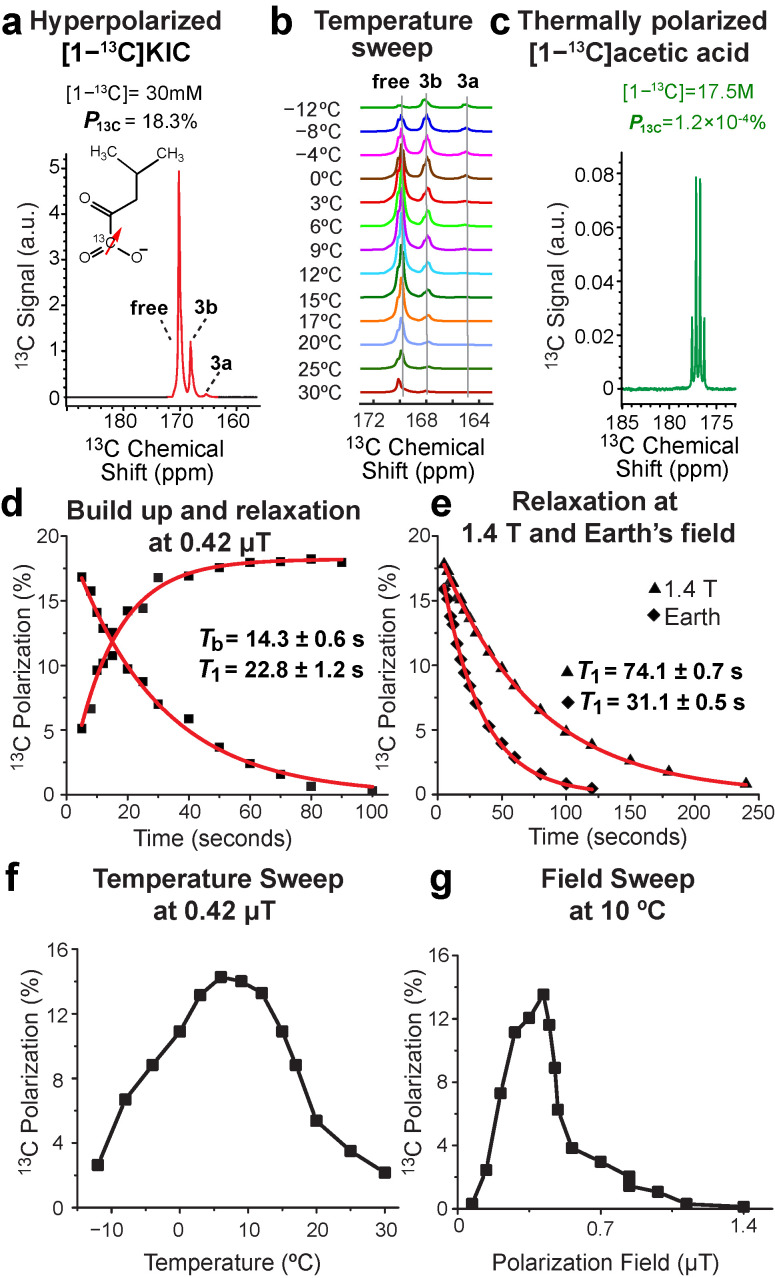
^13^C SABRE-SHEATH studies of [1-^13^C]ketoisocaproate ([1-^13^C]KIC). (**a**) ^13^C spectrum of HP [1-^13^C]KIC. (**b**) Stacked variable-temperature ^13^C spectra showing the interplay between HP complex **3a**, **3b** and the **“free”** resonances for the C1 site as a function of temperature. (**c**) Corresponding ^13^C spectrum of thermally polarized neat [1-^13^C]acetic acid reference sample. (**d**) *P*_13*C*_ build-up and *T*_1_ relaxation at B_T_ = 0.42 μT and 10 °C. (**e**) *T*_1_ at 1.4 T and the Earth’s magnetic field. (**f**) *P*_13*C*_ temperature sweep at B_T_ = 0.42 μT. (**g**) *P*_13*C*_ polarization transfer field (B_T_) sweep at 10 °C.

As expected, the comparative studies with isotopically enriched [1-^13^C]ketoisocaproate ([1-^13^C]KIC) revealed overall the same trends, Figure 2a–g, as those observed for the natural abundance isotopologue, Figure 1. For example, the temperature sweeps (Figure 2b,g), and the B_T_ sweep (Figure 2f) match those for the natural abundance compound, Figure 1.

The detailed quantitative comparison also revealed small differences between the two isotopologues. The maximum *P*_13*C*_ value was 18.3%, Figure 2a, while *T*_b_ and *T*_1_ decay values at 0.42 µT were 14.3 ± 0.6 s and 22.8 ± 1.2 s, respectively, Figure 2d. Moreover, the ^13^C *T*_1_ values at 1.4 T (74.1 ± 0.7 s) and the Earth’s field (31.1 ± 0.5 s) were markedly higher for [1-^13^C]KIC (Figure 2e) compared to those for the natural abundance variant vs. 64.0 ± 1.1 s at 1.4 T and 18.7 ± 0.4 s at the Earth’s field (Figure 1e). We hypothesize that these differences could be potentially attributed to different impurities or other variations in the ketocarboxylate samples—including pH. More detailed studies are certainly warranted to shed more light on the differences in the performance and polarization dynamics of ^13^C-labeled and natural abundance ketoisocaproate samples.

### 3.3. Effect of pH on ^13^C SABRE-SHEATH of [1-^13^C]ketoisocaproate

Prompted by the differences in SABRE-SHEATH efficiency between KIC and [1-^13^C]KIC (see Section 3.2 above for details), a pilot pH-spiking study was performed.

**Figure 3:**
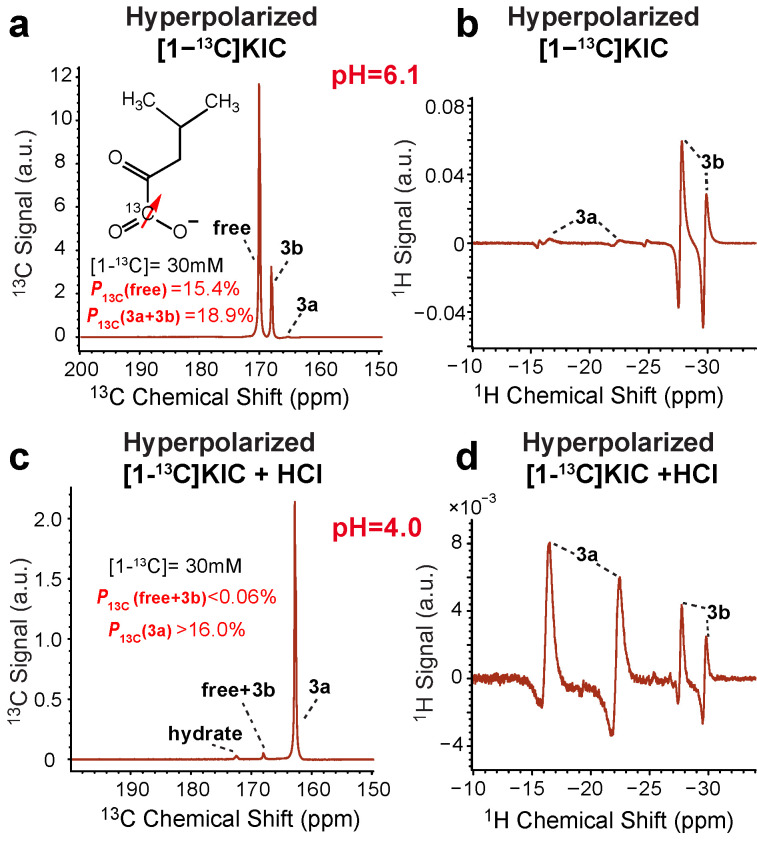
(**a**) ^13^C spectrum of HP [1-^13^C]KIC before adding HCl. (**b**) Hydride region of the corresponding ^1^H spectrum from the HP [1-^13^C]KIC sample before adding HCl. (**c**) ^13^C spectrum of HP [1-^13^C]KIC after adding HCl; (**d**) Hydride region of the corresponding ^1^H spectrum from the HP [1-^13^C]KIC sample after adding HCl. Note that vertical scales are not comparable.

For this pilot study, we added 50 µL of 1 M HCl solution in CD_3_OD (Section 2.6) to the [1-^13^C]KIC (before activation with p-H_2_), which resulted in a pH shift from 6.1 (unaltered solution) to pH 4.0 (acidified sample). At an initial pH of 6.1, we observed *P*_13*C*_ 15.4% and 18.9% for the “**free**” and (**3a + 3b**) species, respectively (Figure 3a). Surprisingly, upon lowering the pH to 4.0, HP species **3a** dominates the ^13^C spectrum shown in Figure 3c. This finding is additionally supported by ^1^H NMR spectroscopy of the hydride spectral region: Figure 3b shows the spectrum of HP [1-^13^C]KIC dominated by **3b** (as expected before the sample acidification), whereas the ^1^H spectrum of the acidified sample is dominated by **3a**. Due to spectral overlap between **3b** and “**free**” resonances in Figure 3c (and by extension ambiguity of the **3a** concentration), *P*_13*C*_ of **3a** was estimated to be in excess of 16.0%. We conclude that pH has a profound effect on the **3a**/**3b** ratio. At lower pH, the equilibrium is shifted to **3a**, which does not exchange with “**free**” species on the time scale of the SABRE-SHEATH experiment (Scheme 1), resulting in no appreciable polarization of the “free” species. Moreover, the acidified sample also exhibited the appearance of the HP ^13^C resonance of the KIC hydrate. Despite this remarkable near-complete disappearance of the HP ^13^C “**free**” species, the polarization transfer remained active in the context of chemical exchange with p-H_2_—as is evident from the high *P*_13*C*_ values of **3a**.

**Figure 4:**
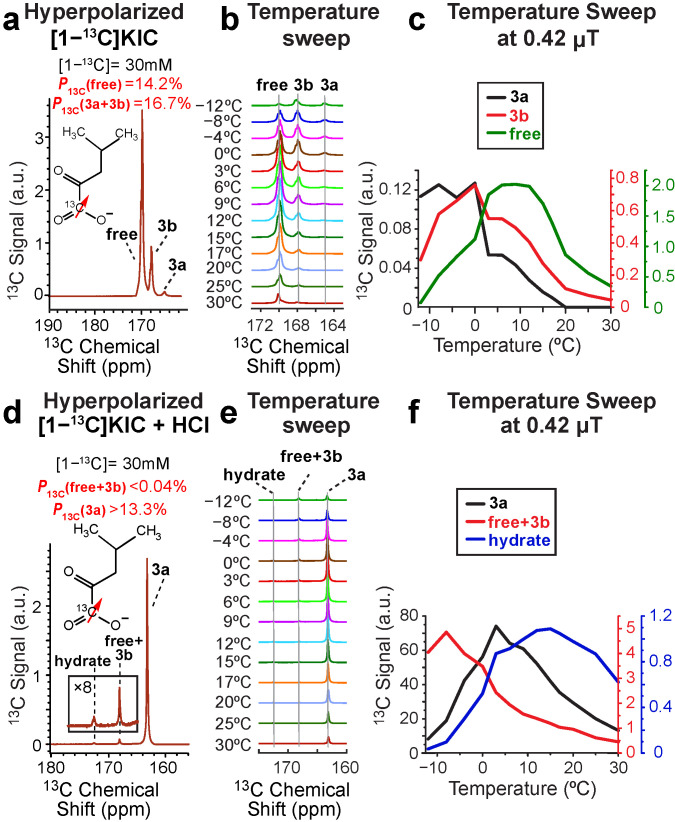
(**a**) ^13^C spectrum of HP [1-^13^C]KIC before adding HCl. (**b**) stacked variable-temperature ^13^C spectra of [1-^13^C]KIC before adding acid, showing the interplay between HP complexes **3a**, **3b** and the “**free**” HP resonances as a function of temperature during the SABRE-SHEATH hyperpolarization process. (**c**) Corresponding temperature sweep projections of HP **3a**, **3b**, and the “**free**” HP resonances from display b. (**d**) ^13^C spectrum of HP [1-^13^C]KIC after adding HCl. (**e**) Stacked variable-temperature ^13^C spectra of [1-^13^C]KIC after adding acid, showing the interplay between HP complex **3a**, **3b**, and the “**free**” HP resonances. (**f**) Corresponding temperature sweep projections of HP **3a**, (**3b** + “**free**”), and the **hydrate** HP resonances from display e.

Prompted by these initial findings, an additional temperature sweep study was performed on a separate sample before and after sample acidification with 50 µL of 1 M HCl solution in CD_3_OD, Figure 4.

The non-acidified sample exhibited similar *P*_13*C*_ values (Figure 4a) to those shown in Figure 3a, demonstrating the overall reproducibility of our sample preparation and hyperpolarization approach. The temperature sweep shown in Figure 4b was also similar to the one presented in Figure 2b. The detailed analysis of the temperature sweep shown in Figure 4c clearly demonstrates that “**free**” species reaches a HP signal maximum at ~10 °C, whereas the maximum for **3a** is at −10 °C, and the maximum for **3b** is at ~0 °C. The temperature sweep performed after sample acidification (Figure 4d,e) reveals that pH also has a profound effect on the maximum temperature positions for the individual HP species. For example, the temperature maximum for **3a** species is shifted to 4 °C. Moreover, the temperature maximum for **3b** + “**free**” (which could not be differentiated due to spectral overlap) is shifted to −8 °C (Figure 4f), which is substantially lower than any of the individual **3b** or “**free**” maxima before the sample acidification (0 °C and 10 °C, respectively, Figure 4c). Therefore, it is concluded that pH modulates not only the relative ratios of **3a** and **3b** (and by extension the “**free**”) HP ^13^C resonances, but it also affects the exchange rates of the substrate with the **3a** and **3b** catalyst species. This finding is important as pH can be potentially employed as a tuning tool to optimize the overall efficiency of the SABRE-SHEATH polarization process. Future detailed studies of this effect are certainly warranted. 

### 3.4. Pilot Post-Mortem ^13^C Dynamic Magnetic Resonance Spectroscopy Using HP [1-^13^C]ketoisocaproate Injection in a Euthanized Mouse

**Figure 5:**
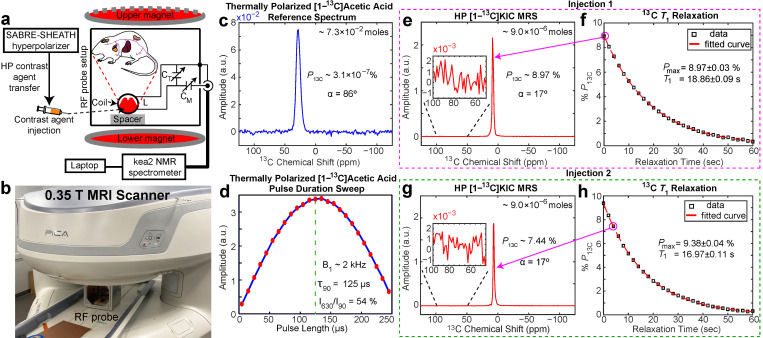
Post-mortem studies employing i.p. injection of HP [1-^13^C]KIC. (**a**) Experimental setup schematic including the circuit diagram of the whole mouse body ^13^C NMR coil used in the work. (**b**) A photograph of the experimental 0.35 T MRI scanner setup. (**c**) ^13^C NMR spectrum from a thermally polarized reference sample of neat [1-^13^C]acetic acid. (**d**) ^13^C RF pulse calibration curve obtained using the thermally polarized reference sample. (**e**) First scan of the ^13^C dynamic MRS run following i.p. injection #1 of HP [1-^13^C]KIC into the euthanized mouse. (**f**) Corresponding ^13^C *T*_1_ decay of the HP signal during dynamic MRS scan #1. (**g**) Third scan of the ^13^C dynamic MRS run following i.p. injection #2 of HP [1-^13^C]KIC into the euthanized mouse. (**h**) Corresponding ^13^C *T*_1_ decay of the HP signal during dynamic MRS scan #2. All *T*_1_ values have been corrected for the effect of excitation RF pulses.

Encouraged by the overall good hyperpolarization potency of HP [1-^13^C]KIC, pilot i.p. injection of HP [1-^13^C]KIC was performed in a freshly euthanized mouse. Our rationale for this experiment was to demonstrate the feasibility of successful injection and detection of HP [1-^13^C]KIC under biologically relevant conditions. To the best of our knowledge, no in vivo demonstration of any HP ^13^C biomolecule has been published using SABRE hyperpolarization technology. Given that the HP solutions were prepared in CD_3_OD, we performed two injections in a freshly euthanized animal, Figure 5.

Before the post-mortem injection, the ^13^C volume detection coil (Figure 5a,b) was optimized using ^13^C detection of a thermally polarized signal–reference phantom (Figure 5c) to determine the optimum RF excitation pulse (Figure 5d). The optimum τ_90_ excitation pulse length was 125 μs, corresponding to a B_1_ strength of 2 kHz, with good B_1_ homogeneity (I_630_/I_90_ = 54%).

Once HP [1-^13^C]KIC was injected into the euthanized animal, the ^13^C detection using 17° excitation pulses started immediately, and dynamic whole body ^13^C MRS was recorded every 2.0 s. The first scan of the first injection is shown in Figure 5e, and the third scan of the second injection is shown in Figure 5g. These ^13^C spectra reveal excellent signal-to-noise ratios (SNRs) in excess of 2000, which can be potentially further boosted by a factor of 3–5 using a singular value decomposition (SVD) approach demonstrated previously [56]. The initial level of *P*_13*C*_ detected inside the animal was 9.0–9.4%, demonstrating good shot-to-shot reproducibility of our HP contrast agent production and administration procedures.

The dynamic ^13^C MRS revealed the *T*_1_ decay of hyperpolarization with mono-exponential rate constants of 18.9 ± 0.1 s (Figure 5f) and 17.0 ± 0.1 s (Figure 5g) for the first and the second injections, respectively. These values are substantially shorter than the corresponding in vitro ^13^C *T*_1_ value of 74.1 ± 0.7 s (Figure 2e) at 1.4 T. Moreover, the post-mortem ^13^C *T*_1_ value is substantially shorter than the ^13^C *T*_1_ in vivo value of structurally similar [1-^13^C]pyruvate (~67 s at 3 T [57]). We rationalize this observation potentially through non-specific interactions between HP [1-^13^C]KIC, residual methanol, the catalyst, and the biological environment. It should be noted that the recent progress with the production of bio-compatible formulations of HP solutions [36] prepared by SABRE may potentially mitigate the ^13^C *T*_1_ reduction challenge. Combined with recent advances in the field of SABRE for purification of HP solutions [58,59,60,61] and the use of heterogeneous [62,63] and aqueous [46,64,65,66,67,68,69] SABRE catalysis, the findings reported here bode well for the future in vivo translation of SABRE-hyperpolarized HP [1-^13^C]KIC.

## 4. Biomedical Outlook

The next logical future steps of the fundamental work presented here in the context of biomedical applications should focus on the validation of SABRE-hyperpolarized [1-^13^C]KIC in animal models of cancer with unregulated BCAT metabolic pathways [22,23]. We envision the feasibility of this approach because the polarization level demonstrated here (*P*_13*C*_ of 18%) is comparable to the in vivo studies that employed the d-DNP hyperpolarization technique. Transitioning to clinical studies using HP [1-^13^C]KIC would require increasing the production scale to that of HP [1-^13^C]pyruvate, which has not been demonstrated yet, representing yet another milestone that must be accomplished in the future.

**Scheme 3:**
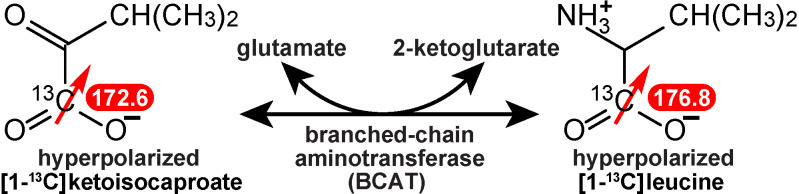
In vivo metabolism of HP [1-^13^C]ketoisocaproate to HP [1-^13^C]leucine. ^13^C chemical shift values are provided in ppm from Reference # [70].

From the experimental standpoint, the in vivo metabolism of HP [1-^13^C]KIC leads to the production of HP [1-^13^C]leucine, which are well separated spectroscopically: 172.6 ppm and 176.8 ppm, respectively, Scheme 3 [70]. It should also be noted that substantially less HP [1-^13^C]leucine is being produced from HP [1-^13^C]KIC compared to that of HP [1-^13^C]pyruvate in many healthy tissues due to the inherently lower metabolic flux of this pathway compared to lactate dehydrogenase (LDH). This difference offers an advantage for clinical applications of a much greater specificity of the HP [1-^13^C]KIC metabolic probe compared to HP [1-^13^C]pyruvate. Indeed, virtually no background signal from intestine tissue was detected compared to murine lymphoma (EL4) tumors [22].

Moreover, previous studies have also revealed that BCAT metabolism may be elevated in some but certainly not in all types and sub-types of solid tumors [22,70,71]. For example, Billingsley and co-workers concluded that metabolic activity in two prostate cancer cell lines (PC3 and DU145) was remarkably low, noting that “the low levels of BCAT activity in the examined prostate cancer cell lines may limit the future application of hyperpolarized [1-^13^C]KIC in the assessment of the disease”. On the other hand, Shu and workers [71] detected high metabolic signal from HP [1-^13^C]KIC in R98 glioma, and Karlsson and co-workers have detected metabolic signal from HP [1-^13^C]KIC in EL4 lymphoma [22]. Furthermore, Butt and co-workers demonstrated the feasibility of employing HP [1-^13^C]KIC for studying cerebral metabolism in a healthy brain [23]. All in all, the pilot small-scale pre-clinical studies employing HP [1-^13^C]KIC as a metabolic probe concluded that HP [1-^13^C]KIC may be an excellent specific probe of BCAT metabolism associated with c-Myc signaling and metastases (moreover, because c-Myc is potentially druggable [72], we envision that HP [1-^13^C]KIC could be a potentially useful metabolic probe for next-generation precision drug development). Therefore, while HP [1-^13^C]pyruvate is an excellent non-specific cancer metabolic probe, we envision that HP [1-^13^C]KIC can provide synergistic information regarding additional profiling in solid tumors. Another area of clinical imaging may involve brain imaging applications—we speculate that HP [1-^13^C]KIC could be potentially useful in the area of brain diseases beyond cancer, including neurodegenerative diseases and aging. Finally, we note that unlike HP [1-^13^C]pyruvate, which is now under evaluation in over 30 clinical trials according to clinicaltrials.gov, clinical studies with HP [1-^13^C]KIC have not been reported yet to the best of our knowledge.

## 5. Conclusions

We have reported the efficient hyperpolarization of KIC and [1-^13^C]KIC and demonstrated *P*_13*C*_ levels of up to 18% with ^13^C *T*_1_ values of up to 74.1 ± 0.7 s. This high degree of polarization is enabled by favorable ^13^C relaxation dynamics in microtesla magnetic fields. It is anticipated that *P*_13*C*_ can be increased further through the use of a higher p-H_2_ pressure [73] and flow rates using more advanced instrumentation. When using an NMR-tube-based experimental setup, a sufficiently high dose of HP contrast agent was produced for future in vivo studies, as demonstrated by pilot experiments in a freshly euthanized mouse. The pilot pH modulation studies revealed remarkable dependence of SABRE hyperpolarization chemistry and dynamics on pH, indicating that pH modulation could be employed as a tuning tool for SABRE-SHEATH hyperpolarization of KIC and other carboxylates, including most notably, pyruvate and α-ketoglutarate.

## Data Availability

NMR spectroscopic data are available from co-authors upon reasonable request. Data is not publicly available due to privacy.
